# Oval Root Canals Prepared With Two Different Endodontic Rotary Files: An In Vitro Study Comparing the Incidence of Dental Defects

**DOI:** 10.7759/cureus.35914

**Published:** 2023-03-08

**Authors:** Bhavika A Bhavsar, Pranjely Sharma, Pratik Surana, Sheetal Badnaware, Dimple Jadhaw, Arvind Jain

**Affiliations:** 1 Department of Conservative Dentistry and Endodontics, RKDF Dental College and Research Center, Bhopal, IND; 2 Department of Pedodontics and Preventive Dentistry, Maitri College of Dentistry and Research Center, Durg, IND; 3 Department of Pedodontics and Preventive Dentistry, Faculty of Dental Sciences, Banaras Hindu University, Varanasi, IND; 4 Department of Conservative Dentistry and Endodontics, Government College of Dentistry, Indore, IND

**Keywords:** tooth wear, dentin, fracture, cracked teeth, sem

## Abstract

Objective: This study aims to investigate and compare the establishment of dentinal cracks during root canal preparation using a stereomicroscope and a scanning electron microscope (SEM).

Materials and methods: Sixty removed human mandibular premolars were separated into three groups of 20: two experimental and one control. Within the randomized controlled experimental groups, root canals were constructed. Group I: the Waldent walflex file and Group II: the Trunatomy (TRN) file. Group III: the control group received no preparations. The surfaces of the roots were checked for dentinal cracks using a stereomicroscope and SEM following sectioning at 3, 6, and 9 mm from the apex. The Chi-square test was used to examine the data.

Results:​​​​​​ In the control group, no fissures appeared. Cracks in Waldent Walflex were almost 66.7% between the 3mm and 6mm range. At both the 6 mm and 9 mm levels, there was no statistically significant difference between the experimental groups (P > 0.05). Defects were found to be substantially higher in the apical region of samples than in the median and coronal sections.

Conclusion: Dentinal fissures were created by every rotary file used in the tests. There were more flaws in the Waldent Walflex file group than in the Trunatomy file group.

## Introduction

Endodontic instruments have come a long way since their inception. In the middle of the 1800s, dentist Edward Maynard developed the first endodontic file [1]. The ease of the material's composition is a major selling point. Hand instrumentation, once a hallmark of endodontic practice but now mostly forgotten, is nonetheless an essential aspect of the canal preparation process. Nickel-titanium (NiTi) instruments were made because stainless steel instruments only clean the root canal system on the surface and can cause problems like ledges, zips, etc. [2].

Human dentin is viscoelastic [3], and during root canal preparations, NiTi rotary instruments on the canals apply rotational forces to the dentin. These rotating files must contact and plane the canal walls to debride the canal. These contacts cause a lot of short-term stress concentrations in the root dentin, which leads to craze lines or tiny cracks. During root canal preparation, stresses are made inside the root canal. These stresses are sent through the root to the surface, where they break the bonds that hold the dentin together. Later could these tiny cracks get bigger and cause a vertical root fracture. This depends on a number of factors, such as the thickness of the root dentine, the strain on the obturation, and the placement of the post [4].

These cracks let bacteria in, which leads to the formation of biofilm and the possibility of re-infection [5]. The apical region, which is important for instrumentation, needs to be cleaned and shaped carefully. But no one has yet recommended the apical preparation size should be [6]. It is also thought that the diameter of the apical preparation affects the number of fractures. Clinicians can make it less likely to break if you keep the canal size as small as possible [7]. Even so, a large apical instrumentation size provides help to reduce debris and bacteria present in the canal [8]. Also, instrumentation that goes all the way to the apical foramen causes more cracks in the apical root than instrumentation that stops short of the apical foramen [9].

Cracks in NiTi files can be caused by heat treatments, designs, the shape of the cross-section, and the way the file moves. Waldent Walflex files (Waldent, India) are rotary files that are very flexible, less likely to wear out from repeated use, better at cutting, have a triangular cross-section, and have sharp cutting edges [10]. Trunatomy (TRN), made by Dentsply Sirona in Switzerland, is a new instrument with different shapes and sizes in its memory and a special way of treating metals. TRN is a rotary file system with an instrument made from 0.8 mm NiTi wire that has been heated in a special way to make it superelastic and less likely to remember its shape [11]. No studies have been done to find out how often cracks in the dentin happen when TRN and Waldent Walflex files are used.

So, the aim of this study was to use a stereomicroscope and scanning electron microscope (SEM) to look at and compare the number of cracks in the dentin after root canal preparation with TRN and Waldent Walflex files. The null hypothesis says that crack formation would be the same in both groups.

## Materials and methods

The present in vitro study was carried out in the Department of Conservative Dentistry and Endodontics and was approved by the Institutional ethical committee bearing approval no: RKDF/DC/PG/2021/17641.

Sixty extracted human mandibular premolar teeth with closed tips and single roots were taken. A periodontal hand scaler was used to clean the teeth and remove soft-tissue debris for 1min. The teeth were disinfected in a 0.1% Thymol-Solution for 24 hours. Roots were coated with silicone impression material (Zhermack Zetaplus, Italy) embedded in an acrylic resin (DPI, India) to simulate the periodontal ligament. Radiographs were taken of the teeth from the mesiodistal and buccolingual sides to rule out the existence of a single canal and single apical foramen with a closed apex. Teeth with root fractures, cracks, open apices, curved canals, multiple roots, caries or fillings, severe anatomical differences, calcified canals, and resorption were not included. Preparing for a root canal with a size #10 K-file (Mani Co, Tokyo, Japan), obstructed canals were discarded, and the working lengths were set at 1 mm short of the apex.

The size #15 K-file made a glide path (Mani Co, Tokyo, Japan). Roots will be set in an acrylic resin that hardens on its own, and a hydrophilic vinyl polysiloxane impression material was used to simulate a Periodontal Ligament (PDL). Based on the different Ni-Ti files used to prepare the teeth, they were randomized into three groups: two experimental and one control (n = 20). Group I: Walden Wal-flex Group III was the control group. Group II was the Trunatomy files.

**Figure 1:**
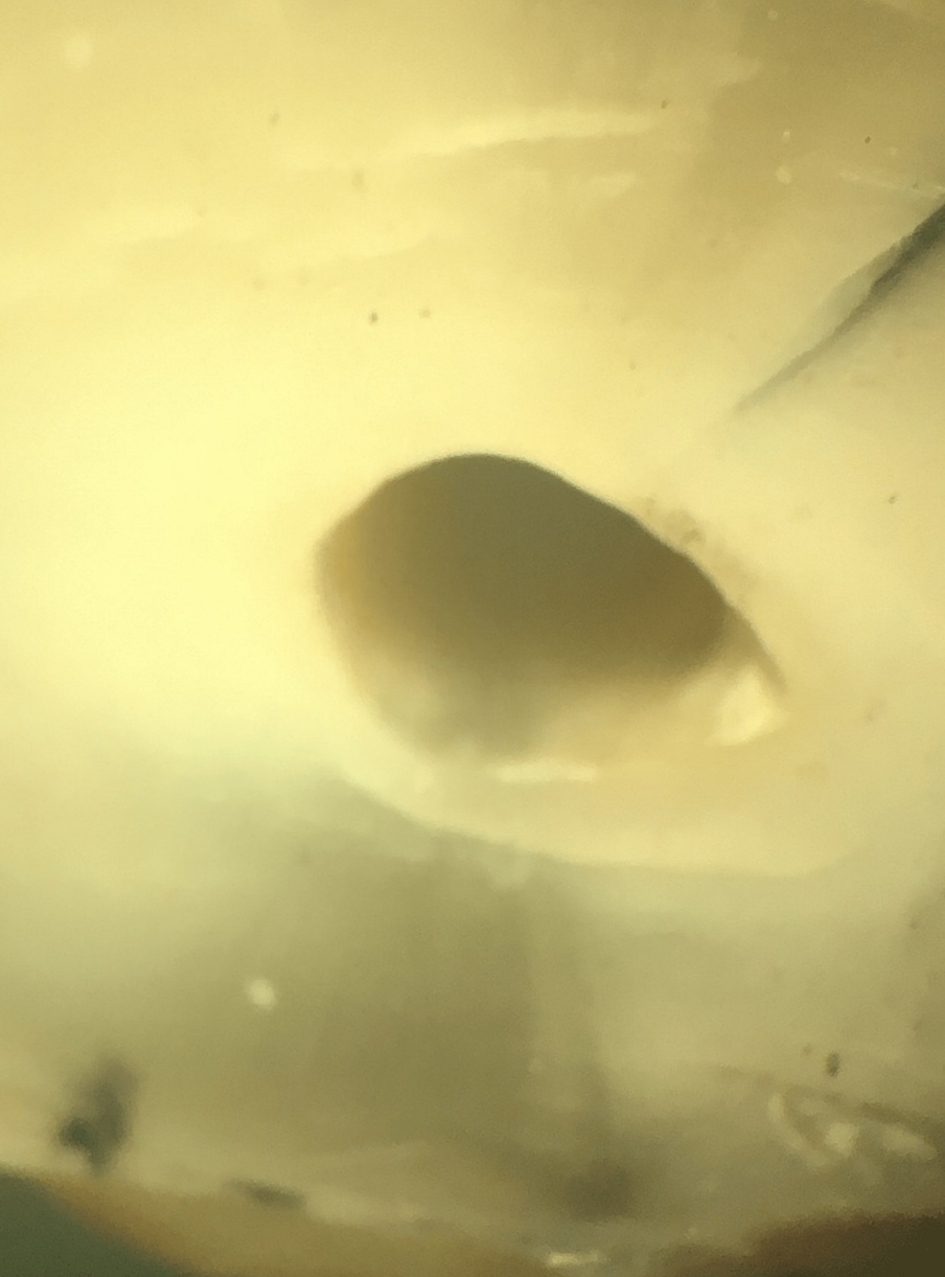
Waldent coronal section after preparation

The canals were disinfected with an Orikam E connected endodontic motor set to the manufacturer's recommended torque and speed of 1.8-3 Ncm and 300 rpm for Waldent Walflex files and 2.5-3 Ncm and 300 rpm for TRN. To avoid bias, all the steps were done by a single, experienced operator. Group I (Waldent Wal flex files) used W1 (17/0.08), W2 (19/0.02), W3 (20/0.04), W4 (20/0.06), and W6 (25/0.06) in order. Group 2 used the TRN orifice modifier (20/0.08), TRN glider (17/0.02), and TRN Prime (26/0.04) until working length. Each file was only used once to make the instruments for one canal. For instrumentation, a full rotation was done with light pecking in and out. In the control group, nothing was done to get ready (Figure 1).

**Figure 2:**
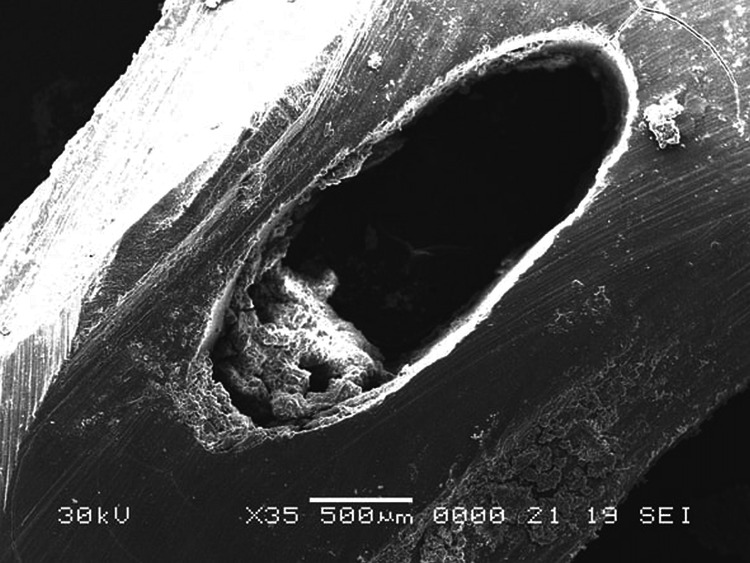
Scanning electron microscopy imaging of the coronal section

The canals were disinfected with 2 mL of 3% sodium hypochlorite (Vishal 5 Dentocare Pvt. Ltd., India), 5 mL of saline (Eurolife, Pirmeera Healthcare Pvt. Ltd., Pune, India), and 5 mL of 17% ethylene diamine tetraacetic acid (Dentwash, Prime Dental, Bhiwandi, India) between each instrument change. The last rinse was with 2 mL. The teeth were decorated with a diamond disc (Addler, Golden Nimbus, Mumbai, India) that was cooled underwater 16 mm from the tip. All of the samples were first viewed under a stereomicroscope (Leica M60; Leica Microsystems GmbH, Wetzlar, Germany) set to 2.5 and the same samples were viewed with a scanning electron microscope (Zeiss Oberkochen, Germany) (Figure 2).

For this study, 60 teeth were chosen that had a single root, a single patent canal, and a closed apex. Dentinal crack evaluation All of the samples were cut with a diamond disc into 3 mm (apical), 6 mm (middle), and 9 mm (coronal) slices from the root apex, perpendicular to the long axis of the teeth. A stereomicroscope and SEM were used to look at the slices. A crack is a flaw that starts in the inner root canal space and spreads to the outside of the tooth. All other flaws were not caused by the canal wall, like craze lines, which were not considered cracks. i) No defect-root dentin free from crazelines or defects on the root surface (inner and outer). ii) Defects- all lines and cracks observed, which were extended to the external root surfaces.

Statistical Package for the Social Sciences (20.0) software was used to analyze the data using the Chi-square test on an Excel sheet (MS Office 2010) (IBM, Chicago, IL, USA). If p is less than 0.05, the result is statistically significant.

## Results

The control group showed no defects at any level. There was a significant difference found amongst the experimental groups (p < 0.05) when compared to the control. When viewed in stereomicroscope the Trunatomy files produced the lesser cracks compared to Waldent Walflex; however, this difference was statistically significant (Table 1).

**Table 1:** Defects in the dentinal cross sections at 3, 6, and 9 (mm) (n=20), viewed in Stereomicroscope % - Percentage, N - Total number

Groups	3 mm	6 mm	9 mm	Total number of specimens presenting defects (%)
Group 1 ( Waldent Wal Flex )	6	4	0	10 (66.7)
Group 2 ( Truanatomy )	4	1	0	5 (33.3)
Group 3 ( Control group )	0	0	0	0 (0.0)
χ^2^ value	6.720	5.673	--	
P value	0.035	0.059	--	

In Table 1, Group 1 (Waldent) shows a total no of 6 cracks at 3 mm and 4 at 6 mm and there were no cracks at the 9 mm level. In Group 2 (TRN) total no of cracks at 3 mm is 4 and at 1 at 6 mm and no cracks are present at 9 mm, respectively. Similarly, when same sections are viewed under the trunatomy files produced a lesser no of cracks when compared to Waldent. The statistical test used in the study is the chi-square test as mentioned above also.

In Group 1 (Waldent) shows a total no of 11 cracks at 3 mm and 4 at 6 mm and no cracks at 9 mm level group. In Group 2 (TRN) total no of cracks at 3 mm are 7 and no of cracks at 6 mm is 2 and no cracks at 9 mm (Table 2).

**Table 2:** Defects in the dentinal cross-sections at 3, 6, and 9 (mm) (n=20), viewed in SEM % - Percentage, SEM - Scanning electron microscope, N - Total number

Groups	3 mm	6 mm	9 mm	Total number of specimens presenting defects (%)
Group 1 (Waldent Walflex)	11	2	0	13 (54.2)
Group 2 (Truanatomy)	7	3	1	11 (45.8)
Group 3 (Control group)	0	0	0	0 (0.0)
χ^2^ value	14.762	3.055	2.034	
P-value	0.001	0.217	0.362	

When looked at through a stereomicroscope, the samples that were cut with the Waldent Walflex had the highest rate of micro-cracks (66.7%), followed by those that were cut with the TRN file (33.3%). When looked at with a SEM, specimens made with the Waldent Walflex had the highest rate of micro-cracks (62.5%), followed by those made with the TRN file (37.5%). But there was no clear difference between the groups (p > 0.05). The chi-squared test showed that the formation of micro-cracks was the same at all section levels (p > 0.05).

## Discussion

In this study, freshly extracted mandibular premolars were used because their small size and thin dentinal walls make them more likely to be affected by forces during instrumentation. The teeth were also easy to get because they were extracted out for orthodontic work and these teeth have closed apex. In this study, researchers examined how TRN files and Waldent Walflex files affect the formation of cracks in the dentin. All of the systems tested in the study caused cracks in the dentin, which is in line with what other studies have found [12,13]. In this study, cracks were found in the dentin of the experimental groups, but not in the control group. So, the null hypothesis could not be verified. Under in vitro conditions, it is not possible to mimic the tooth present in the oral environment. Even though scientists try to recreate clinical settings in the lab, outside factors also play an important role. In this study, the teeth were cut into sections at different heights to look for cracks. When specimens are cut into pieces, cracks can form. In the control group, however, there were no cracks.

In all of the experimental groups, the apical section had more cracks than the middle and coronal sections. This is in line with what Karatas et al. [14], Nishad and Shivamurthy [15], and Chole et al. [16] found in their studies. Stress from repeated instrumentation and the inability of the thin, fragile dentin in the apical area to handle the mechanical stress caused by direct contact with the instrument tip may lead to cracks. The crack could be caused by the shape of the tip, the cross-sectional geometry, the pitch, the taper, and the flute of rotary instruments. More stress is said to be put on canal walls by files that are more tapered (constant or variable) [17]. In this study, however, there were no significant differences between the TRN and Waldent Walflexes when it came to cracking, even though Waldent (W6-25/0.06) has a greater taper than TRN Prime (26/0.04). When compared to Waldent, TRN was determined to have the fewest cracks in this research. This may be because of the three times greater adaptability of the off-center cross section compared to Waldent.

TRN has excellent canal centering capabilities thanks to its particular heat-treated NiTi wire that may be bent before insertion as mentioned by the manufacturers. TRN maintains the integrity of the tooth's structure by removing dentin only when absolutely necessary during a clinical procedure, all the while keeping the canal properly irrigated [11]. At the coronal level, there is no need for excessive enlargement because all of the shaping files are already the same size. Disinfection is guaranteed at the pointy end thanks to the 8-inch diameter shaping files. Irrigation with a 3% Sodium hypochlorite (NaOCl) solution was favored over a 5.25% NaOCl solution since the latter greatly reduces the flexural strength and elastic modulus of human dentin. While the flutes of the file may remain stable at high-stress levels, more cracks were created by Waldent Walflex files, which may be attributable to the robust character of the instrument. Through the use of a stereomicroscope, Elkanti Soujanya et al. [18] compared the number of cracks formed in the dentin following root canal preparation using three different rotary file systems. Microcracking research showed that the ProTaper Next system generated more microcracks than HyFlex and Wave One (P 0.05), but the differences were not statistically significant. When examined by SEM, the apical third of all the file systems had higher instances of cracks than the middle third [19].

The possible limitations of present in vitro study are the sectioning method, difficulty in identifying internal preexisting cracks, and the inability to standardize the speed and torque of both the rotary files used. In literature there is no previous studies to analyze dentinal fracture formation using TRN files. Although the exact etiology cannot be identified, it may affect the durability and robustness of the instruments. Since SEM is a powerful instrument with high magnification and depth of field compared to a stereomicroscope, we have employed it in this investigation after observing using a stereomicroscope.

## Conclusions

Dentinal fissures were created by every rotary file used in the tests. Cracks were more common in the Waldent Walflex files group compared to the TRN files group in both SEM and stereomicroscope images. Defects were found to be substantially higher in the apical region of samples than in the median and coronal sections.
